# Preparation of Parabolic Superhydrophobic Material for Oil-Water Separation

**DOI:** 10.3390/ma11101914

**Published:** 2018-10-09

**Authors:** Xiaoying Qiao, Chunyan Yang, Qian Zhang, Shengke Yang, Yangyang Chen, Dan Zhang, Xiaoyu Yuan, Wenke Wang, Yaqian Zhao

**Affiliations:** 1 Key Laboratory of Subsurface Hydrology and Ecological Effects in Arid Region, Ministry of Education, Chang’an University, Xi’an 710054, China; qiaoxiaoy@163.com (X.Q.); 18829347435@163.com (C.Y.); cherryzhangqian@126.com (Q.Z.); 18291960210@163.com (Y.C.); chzxzdd@126.com (D.Z.); 15129037687@163.com (X.Y.); wenkew@chd.edu.cn (W.W.); 2 School of Environmental Science and Engineering, Chang’an University, Xi’an 710054, China; 3 Engineering Research Center of Groundwater and Ecological Environment in Shaanxi Province, Xi’a 710054, China; 4 Dooge Centre for Water Resource Research, School of Civil Engineering, University College Dublin, Belfield, Dublin 4, Ireland; 2016229046@chd.edu.cn

**Keywords:** superhydrophobic, parabolic structure, surface microstructure, oil-water separation

## Abstract

In order to prepare parabolic superhydrophobic materials, copper meshes were used as the substrate and ultrasonic etching and oxidative corrosion were carried out with FeCl_3_ solution and H_2_O_2_ solution, respectively, and then the surface was modified with stearic acid (SA). The topological structure and surface wettability of the prepared mesh were characterized by fluorescence microscope, scanning electron microscopy and contact angle measurement. Finally, the as-prepared copper meshes were applied to oil-water separation. The results showed that the micro-nano-mastoid structure on the surface of the copper mesh was flaky bulges, forming a rough structure similar to a paraboloid. When the oxidative corrosion time of H_2_O_2_ was 1 min, it is more beneficial to increase the hydrophobicity of the surface of the copper mesh and increase the contact angle of water droplets on the surface of the membrane. Additionally, based on superhydrophobic materials of the parabolic copper mesh, the static contact angles of the water droplets, engine oil and carbon tetrachloride with the surface were approximately 153.6°, 5° and 0.1°, respectively and the sliding angle of the water droplets with the surface were approximately 4.9°. The parabolic membrane was applied to discuss the separation efficiency of different oils with deionized water and the separation efficiency was obtained as benzene > carbon tetrachloride > oil > machine oil. Therefore, based on the research, the parabolic superhydrophobic material has good efficiency of oil-water separation.

## 1. Introduction

In recent years, inspired by the hydrophobic phenomenon of plants and insects, scholars all over the world pay attention to superhydrophobic materials because of their wide application prospects [1,2,3,4]. In order to obtain hydrophobic materials with superior performance, researchers constantly explore the prediction theory of superhydrophobic model and the preparation technology [5,6,7]. In terms of model theory prediction, based on the classical Young’s equation [8], the Wenzel equation [9] and the Cassie-Baxter model [10], in order to explore which structure is more conducive to the superhydrophobicity of the surface, Patankar et al. [11] firstly constructed a cylindrical groove model with micro-nano composite structure and theoretically analyzed the Wenzel equation of the primary structure and the Cassie-Baxter equation under steady state. Yamamoto [12] et al. generalized the microscopic surface into a column for analysis. For modeling and infiltration analysis, Marmur et al. [13] divided the rough surface into a cylindrical structure, a truncated cone structure, a parabolic structure and a hemispherical structure, which verified that the parabolic was most advantageous. Zhang et al. [14] designed the simulated surface microstructure of different models and found that the sinusoidal microstructure similar to the parabolic structure was the most suitable morphology for making superhydrophobic surfaces. It can be seen from the perspective of model analysis that the parabolic structure was the most favorable for forming superhydrophobic materials but there were few reports on how to prepare parabolic superhydrophobic materials. Therefore, the preparation of parabolic superhydrophobic materials was worthy of further exploration.

Based on model predictions, the correct choice of substrate materials was the basis for achieving superhydrophobic surface. In the existing research of superhydrophobic substrate materials, copper is one of the most attractive oil-water separation materials due to its ductility, low density, high specific surface area, mechanical strength, recyclability and environmental friendliness [15,16,17]. Cao et al. [18] found that a 1-dodecanethiol film modified with dopamine was prefabricated on a coarse mesh by a simple impregnation process to obtain a superhydrophobic copper mesh film with a micro-nano layered structure. Rong et al. [16] reported fabrication of a superhydrophobic copper foam with high oil-water separation efficiency, which can serve both as oil absorption material and oil-water separation membrane. In addition, low surface energy is also a key factor in achieving superhydrophobicity, which can be achieved by the introduction of chemical modifiers such as coupling agents and stearic acid. Li et al. [19] constructed a superhydrophobic PVDF/SA nanofiber membrane that maintained high separation efficiency and corrosive treatment after 10 cycles. Fan et al. [20] found that CuO film modified with SA became superhydrophobic and had good corrosion resistance. Maryam Khosravi et al. [21] used a stencil to modify the surface with SA as the substrate and found that it was reused many times during the oil-water separation process without reducing its separation ability. Therefore, the study on the preparation of superhydrophobic materials with copper as the substrate and SA as the modifier have a broad development prospect.

The preparation of superhydrophobic materials was not only related to the substrate material and modifier but also to the construction method. Various methods, including electrospinning [22,23], chemical vapor deposition [24,25] and one-step coating [26,27] have been reported for constructing superhydrophobic membranes with a micro–nano structure [28]. Most of these methods focused on the specific coating technology of hydrophobic materials. Li et al. [19] reported that a facile electrospinning technology was utilized to prepare polyvinylidenefluoride (PVDF)/(SA) nanofibrous membranes. This method is, however, time-consuming and difficult to control accurately. Wu et al. [29] studied the preparation of superhydrophobic surfaces by spraying, dipping, painting and so forth, and used nanoparticles (i.e., SiO_2_ or TiO_2_) and epoxy resin as raw materials to simulate ordinary household coatings, which was easy to operate but the surface stability and the durability of the modification layer were often too poor. In order to improve these problems, wet etching [15,30,31] technology has been developed in recent years. Cao and co-workers [32] fabricated super-wettable surfaces on copper mesh and copper foam by etching with H_2_O_2_ and HNO_3_ and then immersed them into AgNO_3_ solution, the Cu@Ag films were formed on copper substrates and exhibited superhydrophilicity and underwater superoleophobicity. This method is more effective for constructing a surface roughness structure, however, the etching solution is often harmful to the environment. Similar research results were [33,34] and so forth, they only paid attention to the roughness of the surface of the material and there was very little analysis on how to etch a specific morphology. Therefore, the technique used to etch a specific shape on the surface and then apply it as a superhydrophobic material was the most interesting issue.

In this study, a 200 copper mesh was used as the substrate, firstly, the copper mesh was ultrasonically etched to roughen the surface and then ultrasonic oxidized and etched to realize the parabolic specific morphology. The copper mesh was modified with SA to obtain the surface of the copper mesh similar to the parabolic rough structure. Then, the superhydrophobic surface was characterized by fluorescence microscopy, scanning electron microscopy (SEM) and wettability. Finally, the parabolic copper mesh was applied to oil-water separation and the relationship between superhydrophobic surface contact angle and oil-water separation efficiency was studied for different density oils.

## 2. Experiment

### 2.1. Reagents and Materials

200 Copper mesh, acetone, ethyl alcohol, stearic acid, ferric chloride, hydrogen peroxide (30 wt%), deionized water, benzene, cooking oil, engine oil, carbon tetrachloride. All other reagents are analytical grade and used as received. All chemical reagents were purchased from Tianjin Komi Chemical Reagent Co., Ltd. (Tianjin, China).

### 2.2. Fabrication of Parabolic Superhydrophobic Material

First, 200 copper meshes with a size of 4 cm × 4 cm were ultrasonically cleaned using acetone, ethanol and deionized water for ten minutes sequentially, to remove any impurity residual on its surface. After cleaning, the copper meshes were dried with N_2_. Then the copper meshes were chemically etched using 35%FeCl_3_ solution for 20 min in an ultrasonic bath. After that, the etching samples were immersed into the 30%H_2_O_2_ solution for different times (0 min, 1 min, 2 min and 5 min) to deposit a uniform layer of oxidation film on samples surface, respectively. And then the samples were rinsed with distilled water and dried with N_2_. Finally, the as prepared copper meshes were immersed in a 10 mmol/L ethanolic/water solution (at a volume ration of 1:3) of SA for 30 h at 60 °C. The resulting membranes were rinsed with ethanol and deionized water and then dried at room temperature. The obtained copper meshes were subjected to further characterizations.

### 2.3. Characterization

#### 2.3.1. Fluorescence Microscope Characterization

The prepared hydrophobic mesh membrane was placed in the center of the slide and placed flat on the stage of the fluorescence microscope (LW300LFT, Xi’an Weiwei Optoelectronic Technology Co., Ltd., Xi’an, China). The objective lens was adjusted to PL100/1.25 (0.17) and the eyepiece magnification was 10×. And then the changes on the surface morphology of the copper mesh were observed.

#### 2.3.2. SEM Characterization

The surface morphologies of the copper meshes obtained in different solutions were characterized by SEM imaging on a FEI Quanta 200 SEM (Hillsboro, OR, USA). It is necessary to carry out gold spray on the surface of the sample before the test, which can well observe the microscopic morphology of the copper mesh surface and enhance the conductivity of the sample and then the sample was fixed on the sample stage to observe. All steps were carried out in a vacuum environment.

#### 2.3.3. Contact Angle Measurements

The contact angles (CAs) of the deionized water, engine oil and carbon tetrachloride and the sliding angle of the deionized water were measured at ambient temperature using a SL200KS contact angle meter (provided by American KINO Industry Co., Ltd., Somerville, Boston, MA, USA). The volume of the test water droplet was approximately 3 μL. At least three parallel positions on the surface were measured to obtain the average contact angle values and the accuracy was 0.1°.

### 2.4. Oil-Water Separation Test 

The as-prepared superhydrophobic copper mesh was fixed in homemade oil-water separation system. Oils of different densities (Sultan I staining) and deionized water (methylene blue staining) were mixed at a volume ratio of 1:1, respectively. Carbon tetrachloride (density greater than water) and benzene (density less than water) were mixed with deionized water respectively to separate oil from water under the driving force of gravity. After the separation of oil-water, the oil in the beaker will be recycled and the selective separation process was recorded with a digital camera. According to the oil volume of the meshes, the oil-water separation efficiency was calculated by the following formula.

(1) $$\eta =\frac{V_{R}}{V_{0}}\times 100\%$$

where *V_R_* is the recovered oil volume and *V*_0_ is the initial oil volume.

## 3. Results and Discussion

### 3.1. Fluorescence Microscope Characterization

**Figure 1:**
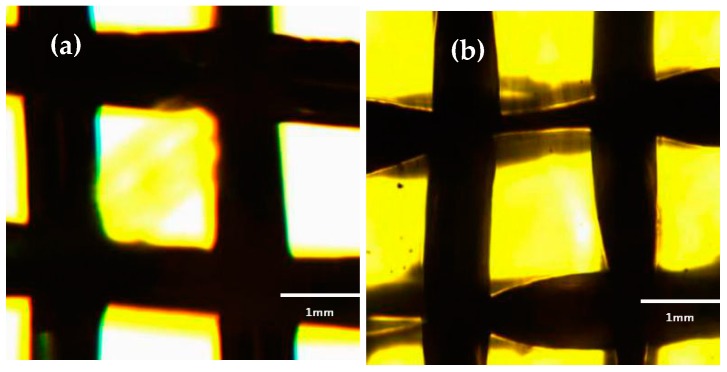
Copper mesh morphology under fluorescence microscope. (**a**) Untreated copper mesh; (**b**) Etching and oxidation copper mesh.

The morphology of copper mesh before and after etching and oxidation under fluorescence microscope was shown in Figure 1. As can be seen that the shape of the copper mesh had a significant change. The size of the untreated copper mesh was uniform and the thickness of the copper mesh was basically the same. However, the copper mesh after etching and oxidation was clearly narrowed, indicating that part of the mesh of the copper mesh was subjected to cavitation impact through the ultrasonic etching of the acidic etchant FeCl_3_ solution, the surface of the copper meshes had been deformed and the materials had been eroded. That is, the cavitation erosion occurred. This ultrasonic cavitation and cavitation can enhance the etching effect and form a three-dimensional rough surface on the surface of the copper mesh, which is a necessary condition for oil-water separation [35]. Therefore, the treatment of copper meshes with FeCl_3_ and H_2_O_2_ solutions was crucial for the formation of superhydrophobic surfaces.

**Figure 2:**
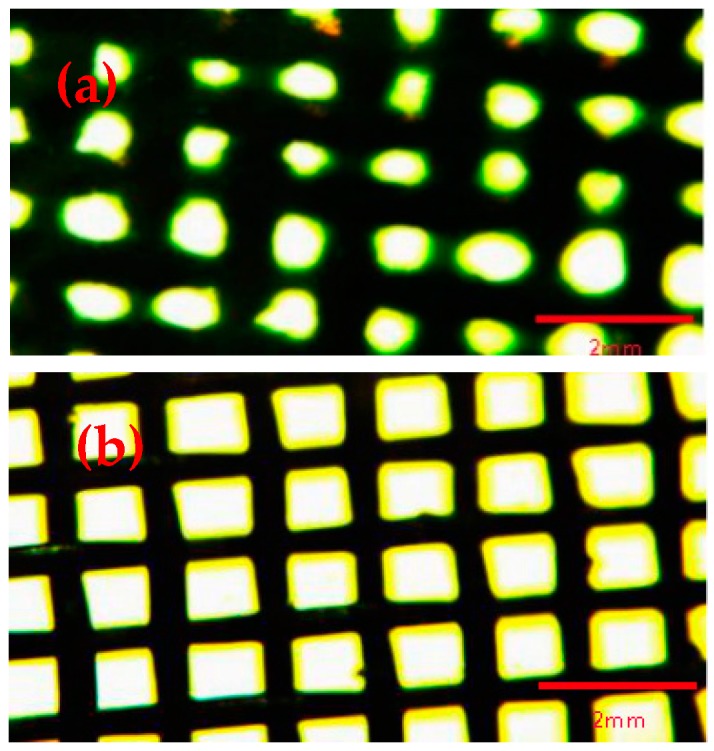
(**a**) Copper mesh modified by SA after etching and oxidation; (**b**) Etching of copper mesh without modification of SA after oxidation.

Figure 2 showed that the surface morphology of copper mesh after etching and oxidation without modification and modification of SA. By comparing Figure 2a and Figure 2b, it can be seen that after the copper mesh was modified with SA, the pore diameter of the mesh became significantly smaller, which indicated that SA was successfully attached to the surface of the copper meshes and the copper mesh surface became rough, indicating that the surface roughness of the copper mesh was increased by modification with SA [19,36]. However, the meshes were still present, so the separation effect will not be affected when the screen hole was blocked by SA.

### 3.2. SEM Characterization

Owing to the fact that the fluorescence microscope can only roughly see the difference in surface morphology of the copper mesh before and after treatment. Thus, we used a SEM to characterize the sample for further observed the microstructure of the copper mesh surface.

#### 3.2.1. Effect of Oxidation Time of Hydrogen Peroxide

**Figure 3:**
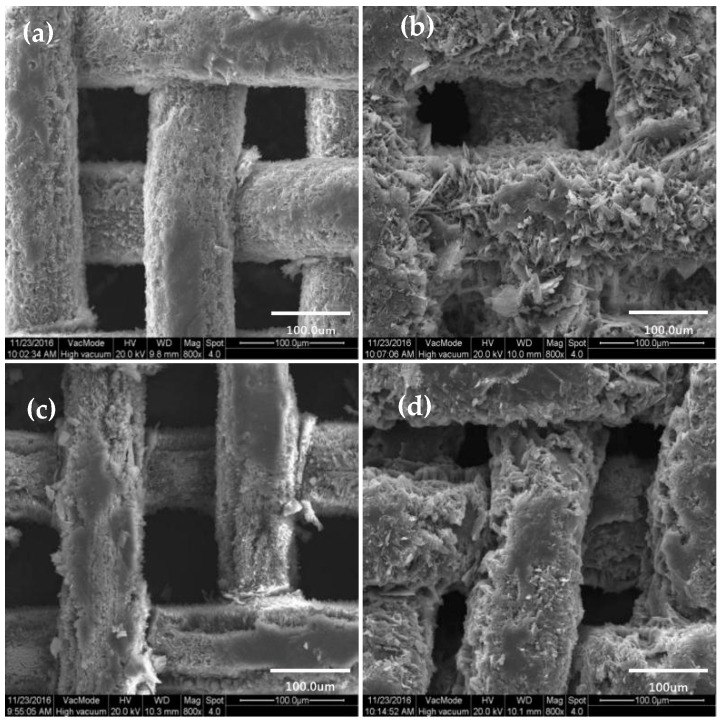
SEM image of copper mesh modified SA at different oxidation times. (**a**) oxidation time 0 min; (**b**) oxidation time 1 min; (**c**) oxidation time 2 min; (**d**) oxidation time 5 min.

After modification of SA by copper mesh with different oxidation time, the surface morphology was characterized by SEM, as shown in Figure 3.

It can be clearly seen from the Figure 3a that when the oxidation time was 0, the surface of the copper mesh after the SA modification did not show large irregular mastoid structure. However, when the oxidation time of hydrogen peroxide was 1min, as shown in Figure 3b, the surface of the copper mesh was scattered with irregular sheet mastoid which were basically consistent in height. Meanwhile, Rong et al. [1] also studied the amplified SEM images in the three-dimensional copper foam, which showed papillae with a flower-like structure. These micro-nano-blocked mastoids of different sizes formed mastoid clusters and there were obviously spacing and gap between the blocky mastoids and the mastoid clusters, which can store air. Therefore, this can form the air cushion on the surface of the copper mesh to prevent contact between the water droplets and the solid surface, which increased the CAs of the water. With the increase of oxidation time, as shown in Figure 3c,d, it can be seen that the mastoid clusters on the surface of the membrane showed a large area of massive structure and the spacing and gap between the mastoids and the mastoid clusters became smaller. When the oxidation time of hydrogen peroxide was 5 min, the mastoid clusters on the surface of the membrane were more compact. At this point, the space between the membrane surface can be used to store air was small, which was not conducive to improving the hydrophobicity of the membrane surface.

According to the study of the hydrophobic surface structure of the lotus leaf and the Cassie-Baxter theory, the air cushion on the surface of the membrane was essential for obtaining a hydrophobic surface [10,16,37,38]. From the comprehensive analysis of Figure 3, it was more beneficial to obtain a rough structure similar to the geometry of the lotus leaf surface when the oxidation time of hydrogen peroxide was 1 min. Therefore, the selection of hydrogen peroxide oxidation time was 1 min.

#### 3.2.2. Surface Morphology of Copper Mesh Not Modified by SA

**Figure 4:**
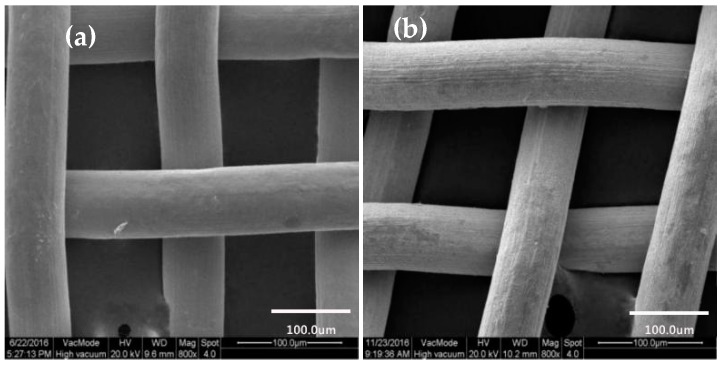
SEM image of copper mesh at 800×. (**a**) Surface morphology of untreated copper mesh; (**b**) Surface morphology of copper mesh after etching and oxidation.

Figure 4a,b were SEM images of untreated and etching and oxidation copper meshes with the 800× without SA modification, respectively. Comparing Figure 4a with Figure 4b, it showed that the surface morphology of the etching and oxidation copper mesh had changed significantly. The surface of the untreated copper mesh was smooth, while the etching and oxidation copper mesh was basically unchanged in shape and the surface of the copper mesh became very rough [39]. In addition, the diameter of copper mesh wire was slightly narrowed and the corresponding mesh size was slightly increased, which was basically consistent with the observation under the fluorescence microscope, indicating that copper mesh was affected by ultrasonic cavitation and cavitation after ultrasonic etching of acidic etching agent FeCl_3_ solution and even rough morphology was formed on the surface of copper mesh.

#### 3.2.3. Surface Morphology of Copper Mesh Modified with SA

**Figure 5:**
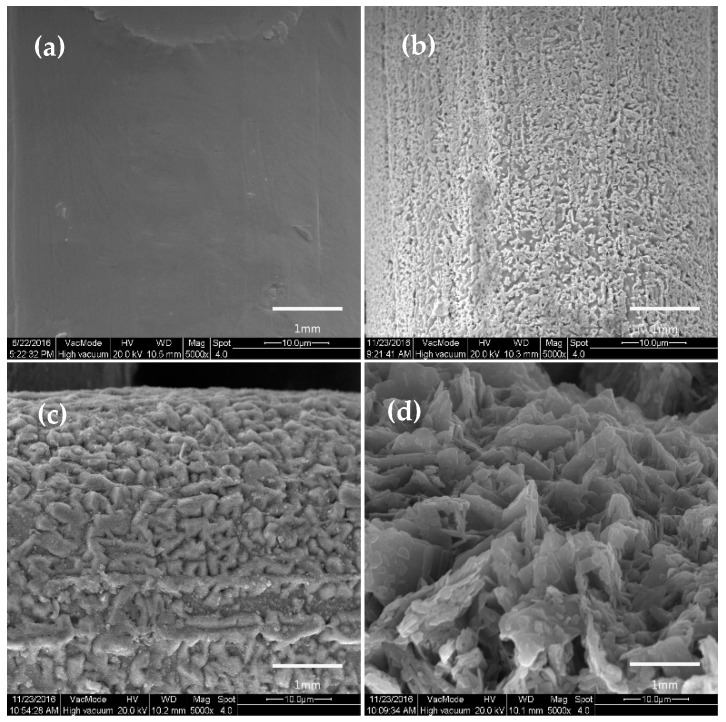
Copper mesh surface morphology. (**a**) Untreated copper mesh; (**b**) Etched copper mesh in FeCl_3_ solution; (**c**) Etching and oxidation copper mesh; (**d**) Copper mesh modified with SA.

The untreated copper mesh and the morphology of the copper mesh under different treatment processes were shown in Figure 5.

As can be seen from Figure 5a, the surface of the untreated copper mesh in the SEM at 5000× was smooth. And after ultrasonic etching by FeCl_3_ solution, the surface of the copper mesh became rough, showing a microporous structure of different sizes as shown in Figure 5b. Comparing Figure 5b and Figure 5c, it can be concluded that after H_2_O_2_ solution oxidation etching, there was a large number of mastoid structures on the surface of the copper mesh and the surface roughness was obviously improved. Figure 5d showed that the surface of the copper mesh was completely covered with SA after modification with SA, compared with Figure 5c, the surface mastoid structures were interlaced and clustered to form a large number of mastoid clusters, which further improved the surface roughness of the copper mesh.

**Figure 6:**
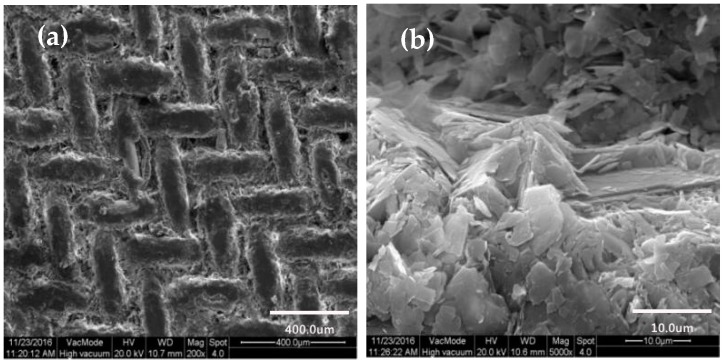
SEM image of the surface morphology of the etching and oxidation copper mesh after modification of SA. (**a**) at a low magnification (200×); (**b**) at a high magnification (5000×).

Figure 6 showed the surface morphology of the etching and oxidation copper mesh after modification of SA.

Under 200× electron microscopy, as shown in Figure 6a. After the copper mesh was modified with SA, the surface of the copper mesh substrate was uniformly adhered by SA and it was apparent that the surface after the modification was a rough surface, moreover, the mastoid height was basically the same. By contrast, a 5000× SEM was shown in Figure 6b. The micro-nano-mastoid structure on the surface of the copper mesh modified by SA was a sheet-like bulge and adjacent sheet mastoids were interlaced and clustered to form a mastoid cluster similar to a paraboloid. In addition, there were clear spacing and gap between the mastoid clusters and these voids can capture a large amount of air, which had a significant impact on the hydrophobic properties of the surface. Therefore, it can be concluded that after modifying the SA, the copper mesh can form a rough structure similar to a paraboloid.

**Figure 7:**
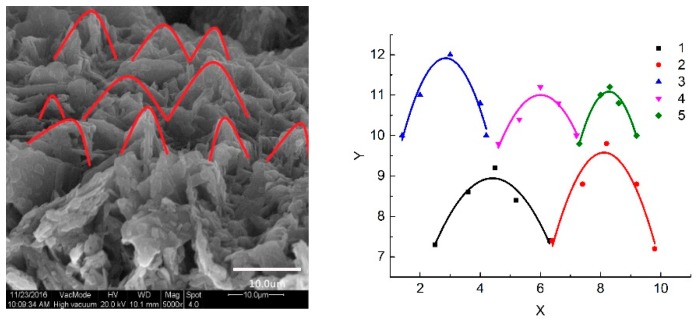
Microscopic morphology fitting curve of membrane SEM.

In order to further prove that the surface morphology of the copper mesh was parabolic, the microstructure of the copper mesh surface in the SEM image was generalized, as shown in Figure 7.

It can be seen that the fitting curve obtained from the SEM image was parabolic and the correlation coefficient was greater than 0.80 (as shown in Table 1). According to the absolute value of a in Table 1, the opening of the first parabola was the largest and the fifth was the smallest, it meant that a parabolic micro-nano structure of various sizes was formed on the surface of the copper mesh. Therefore, the copper mesh modified by SA can form a rough structure similar to a parabolic structure.

### 3.3. Wettability Characterization

The CAs of a droplet on a solid surface a direct measure of its wetting performance. For lyophobic surfaces, the droplets were not easily spread and appeared spherical on the surface, however, for lyophilic surfaces, the droplets were easily wet the surface and spread out.

**Figure 8:**
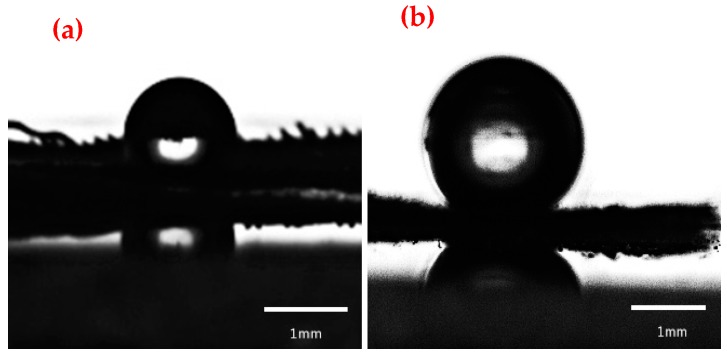
Wettability of water droplets on the surface of the membrane. (**a**) Untreated copper mesh; (**b**) As-prepared copper mesh.

The wetting behaviors of water-oils on the as-prepared copper meshes were evaluated by the contact angle measurement. The CA of the water droplets on the untreated copper mesh and the as-prepared copper mesh were shown in Figure 8a,b. The untreated copper mesh was hydrophilic and the CA of water droplets with the surface was 86.3°, however, the static contact angle of the water droplets with the surface of the as-prepared copper mesh was measured to be 153.6°. That is, the surface of the prepared copper mesh which had a parabolic rough structure was a superhydrophobic surface. Cao et al. reported that the DDT-modified copper mesh showed a superhydrophobicity and the CA of water droplets with the surface was 152° [32]. Therefore the hydrophobic properties of the copper mesh modified with SA had certain advantages.

**Figure 9:**
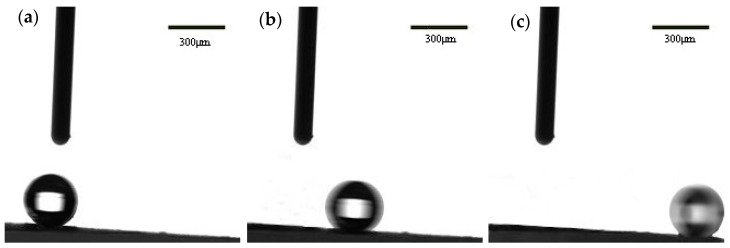
The diagram of water droplets (drop volume of 3 μL) sliding on the surface of the as-prepared copper mesh. (**a**) The initial state of the water drops; (**b**) The rolling state of water droplets; (**c**) The rolling state of water droplets at the bottom of the slope.

It is well known that the static contact angle and the dynamic contact angle (sliding angle) are important indicators for measuring the wetting properties. When the static contact angle is greater than 150° and the dynamic contact angle (sliding angle) is less than 10°, it can be proved that the materials have good superhydrophobic properties. The diagram of water droplets sliding on the inclined surface was shown in Figure 9. It can be seen that when the measuring device is inclined at a certain angle, the water droplet can freely slide down along the inclined surface from the Figure 9. After five tests, the critical angle (sliding angle) of the water droplets slipped instantly was measured to be approximate 4.9°. Therefore, it can be concluded that the copper mesh with a parabolic structure is superhydrophobic.

**Figure 10:**
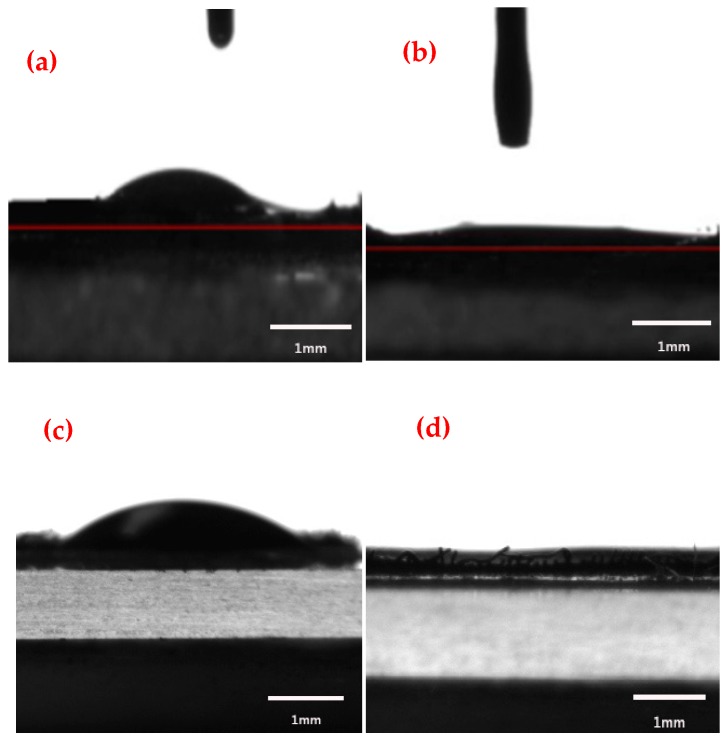
Wettability of oil droplets on the surface of the membrane. (**a**,**c**) Untreated copper mesh; (**b**,**d**) As-prepared copper mesh.

Analogously, the CAs of the engine oil and carbon tetrachloride on the untreated copper mesh and the as-prepared copper mesh were measured, as shown in Figure 10a–d. Among them, ab and cd showed the wettability of engine oil and carbon tetrachloride on the surface of the two kinds of membranes, respectively. After taking the average of three measurements, the CA of the engine oil on the surface of the untreated copper mesh was measured to be approximate 60.2°, as shown in Figure 10a, indicating that the copper mesh membrane was inherently lipophilic [1,40]. Figure 10b showed that the modified copper mesh surface had a super-lipophilic property and the CA with the engine oil was approximate 5°, which indicated that the copper mesh with the rough parabolic structure was superhydrophobic surfaces.

Because carbon tetrachloride had a much lower viscosity than engine oil, carbon tetrachloride was immediately spread on the surface of the prepared copper mesh in the experiments and the CA that was approximate 0.1° was obtained as shown in Figure 10d. Analogously, the CA of the untreated copper mesh surface was approximate 67.4°, as shown in Figure 10c. Comparing Figure 10c with Figure 10d, it can conclude that the as-prepared copper meshes with the parabolic microstructure was super-lipophilic when the oil droplet was carbon tetrachloride. Comparing Figure 10b and Figure 10d, because the oil type was different (i.e., oil/carbon tetrachloride, light oil/heavy oil), the viscosity coefficient was different, resulting in different CAs of oil droplets on the surface of the material.

In summary, by measuring the wettability of water droplets, engine oil and carbon tetrachloride on two kinds of membranes, it was shown that the microscopic morphology of the surface of the material directly affected the CAs, moreover, the hydrophobic and lipophilic properties of the material affected by its CAs. So, the copper mesh surface with the microstructure of the paraboloid was the superhydrophobic surface.

### 3.4. Determination of Oil/Water Separation Performance

**Figure 11:**
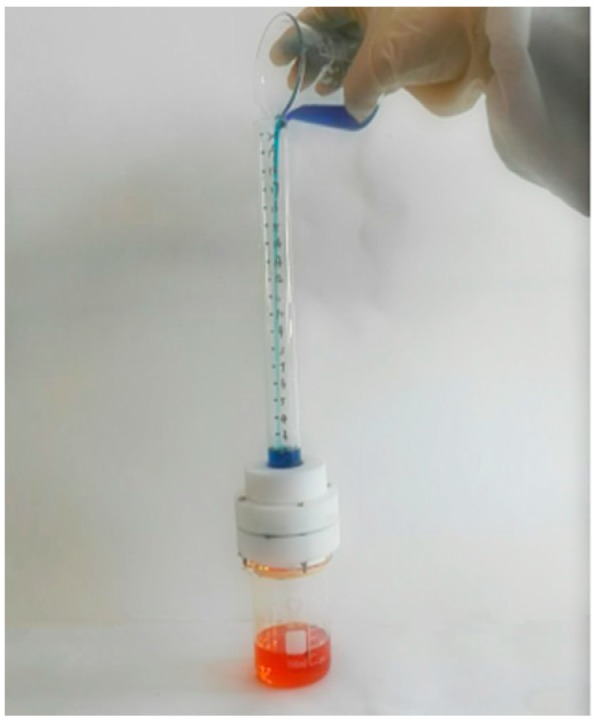
Oil-water separation device.

Figure 11 showed the oil-water separation process after mixing 30 mL of carbon tetrachloride with 30 mL of deionized water.

In the oil-water separation process, the density of carbon tetrachloride was greater than that of water, so the blue deionized water firstly entered the separation device through the upper glass tube. As mentioned above, the as-prepared membrane was superhydrophobic, the deionized water was all the time blocked on the membrane, red carbon tetrachloride, meanwhile, through the blue deionized water reached the surface of the membrane and the red carbon tetrachloride penetrated downward through the membrane under the action of gravity and capillary force, then gradually flowed into the beaker below the device, thereby the separation of oil-water was achieved. Analogously, the oil-water separation experiment of benzene was carried out.

#### 3.4.1. Separation Efficiency of Different Oil-Water Mixtures

**Figure 12:**
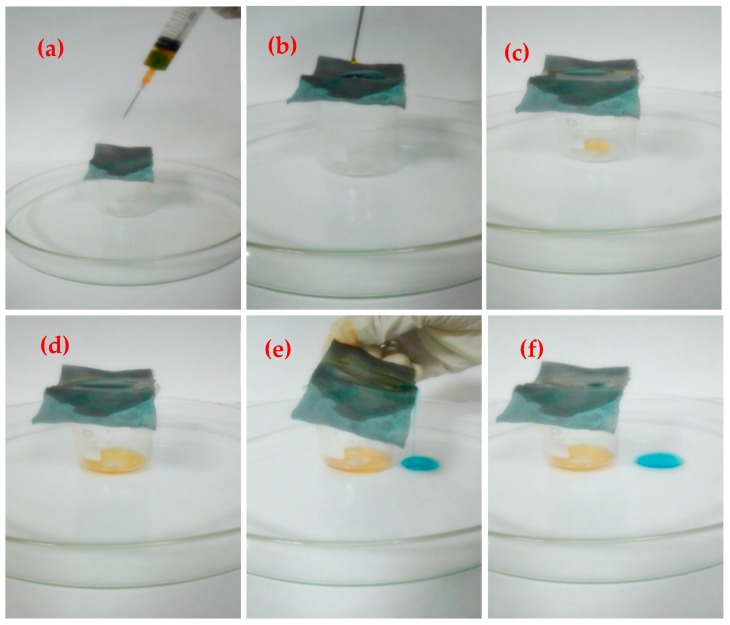
Separation process of cooking oil and water mixtures. (**a**,**b**) Oil-water separation operation; (**c**,**d**) Oil-water separation process; (**e**,**f**) Operation after separation of oil-water.

The density of engine oil and cooking oil is less than that of water and the viscosity of engine oil and cooking oil is much higher than the carbon tetrachloride and benzene, thus, it was not effective to separate the engine oil and the cooking oil using the above device. In order to accurately measure the separation efficiency of the copper mesh for different oil-water mixtures, the oil-water separation experiment was carried out by using a self-made simple oil-water separation device. Taking the cooking oil as an example in Figure 12.

As shown in Figure 12a, the oil-water mixtures prepared from 4 mL of engine oil and 2 mL of deionized water in a syringe and the prepared membrane was placed on top of the small beaker to completely cover the mouth of the beaker and the small beaker was placed in the center of the large culture dish. After shaking the mixture of oil and water in the syringe, then gradually squeezed the syringe and slowly dripped the mixture from the needle to the membrane, as shown in Figure 12b. Due to gravity and capillary forces, as well as the superlipophilic properties of the copper mesh, the oil droplets gradually flowed through the membrane into the small beaker. However, because of the superhydrophobicity, water was trapped above the membrane, as shown in Figure 12c,d. The process of oil-water separation in the whole process was approximately 4 min. Figure 12e,f showed that after finishing separating the oil-water, the water that trapped above the copper mesh was poured into a large petri dish and almost no blue residue was observed on the membrane.

**Figure 13:**
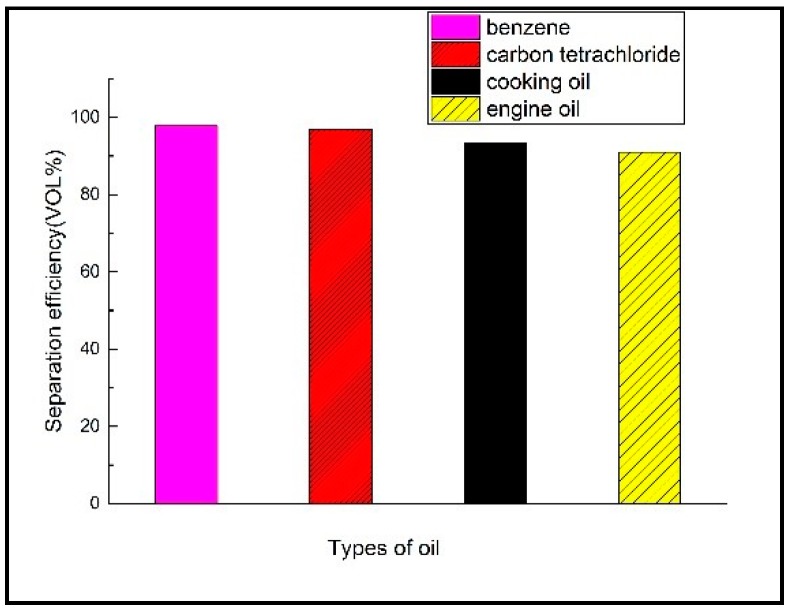
Separation efficiency diagram of different oil-water mixtures.

According to the above methods, the mixtures of engine oil, benzene, carbon tetrachloride and deionized water were separated by oil-water separation experiment and the oil-water separation efficiency of these four different oils was measured, respectively. The results were shown in Figure 13.

It can be seen from Figure 13 that the separation of benzene, carbon tetrachloride, cooking oil and engine oil by the prepared parabolic morphology copper mesh was more than 91% and even the separation efficiency of benzene and carbon tetrachloride mixture was above 97%. Moreover, the separation efficiency of the oil-water mixture of cooking oil and engine oil was clearly smaller than the benzene and carbon tetrachloride. It can be seen that due to different oil types and different parameters such as viscosity coefficient, thus, the CAs of the oil droplets on the surface of the membrane were different and the separation efficiency was also different [21,41,42]. Hereby a comparison of the copper mesh prepared from different modified materials and methods for oil water separation is summarized in Table 2. And the separation efficiency and oil-water mixtures were shown in Table 2.

It can be seen from the Table 2 that the parabolic superhydrophobic material prepared in this study had certain advantages for oil-water separation and the etching method was more advantageous than other methods. In addition, different modified materials, methods and oils have an effect on the separation efficiency.

#### 3.4.2. Relationship between CAs and Oil-Water Separation Efficiency

The oil-water separation efficiency is not only related to the viscosity coefficient of oil droplets but also closely related to the contact angle. For this reason, the separation efficiency of different oils and CA of the copper mesh with the rough shape of the paraboloid were experimentally determined and the separation efficiency was determined by a needle tube experiment, the whole oil-water separation process after mixing 4mL benzene with 2 mL deionized water lasted approximately 15 s and the separation process of the same dose of carbon tetrachloride, cooking oil, engine oil took about 20 s, 4 min 5 s, 5 min 44 s, respectively and the average rate of decline of oil droplets was engine oil < cooking oil < carbon tetrachloride < benzene, the aforementioned oil-water separation efficiency was benzene > carbon tetrachloride > cooking oil > engine oil, therefore, the oil and water separation efficiency was positively correlated with the average flow rate of oil droplets. The contact angle of the oil with the membrane was benzene (0°) < carbon tetrachloride (0.1°) < cooking oil (4.6°) < engine oil (5°), which can be obtained: (a) The smaller the contact angle of the surface of the membrane, the larger the average flow rate of the oil droplets, the conclusion verified the rule that the CAs was positively correlated with the average flow velocity of water droplets and negatively correlated with the average flow velocity of oil droplets by Zhang [47]. (b) The smaller the CAs of the surface of the membrane, the higher the separation efficiency of oil-water [48].

## 4. Conclusions

In a summary, by controlling etching and oxidation conditions, the rough micro-nano structure can be obtained on the surface of the copper mesh and finally a parabolic superhydrophobic membrane was obtained by modifying the SA with low surface energy. The as-prepared copper mesh was used for the oil-water separation of different oils and the oil-water separation efficiency was benzene > carbon tetrachloride > cooking oil > engine oil, therefore, the parabolic superhydrophobic membrane has a good oil-water separation effect.

## Figures and Tables

**Table 1:** Fitted parabolic equation parameters.

Serial Number	a	h	k	R^2^
1	−0.44935	4.4073	8.9446	0.89199
2	−0.78860	8.1127	9.5793	0.89229
3	−0.96367	2.8509	11.9148	0.91121
4	−0.66353	6.0041	11.0044	0.80343
5	−1.32367	8.2791	11.0860	0.95173

Equation: $y=a\times {\left(x-h\right)}^{2}+k$. The equation is the apex of the quadratic function which is transformed from the general form of the fitted curve equation, where x, y represent the independent and dependent variables of the quadratic function, respectively.

**Table 2:** Comparison of the copper mesh membranes materials for oil/water separation.

Year	Matrix	Modified Materials	Method	Separation Efficiency (%)	Oil-Water Mixtures	Ref.
2018	Cu mesh	Stearic acid (SA)	etching	>97	benzene, carbon tetrachloride	This work
2018	Cu mesh	dodecanethiol (DDT)	etching	>98	cyclohexane, n-hexane	[32]
2017	Cu mesh	Candle soot	Deposition	95	–	[35]
2017	Cu mesh	CuWO_4_@Cu_2_O	Electrochemical anodization	95	cyclohexane, chloroform	[43]
2017	Cu mesh	Cu_2_S@Cu_2_O	Chemical bath deposition	>94	isooctane, chloroform	[44]
2017	Cu mesh	Dopamine; 1-dodecanethiol	Dip-coating	90	cyclohexane, n-hexane	[18]
2017	Cu mesh	1-dodecanethiol (HS(CH_2_)_11_CH_3_)	etching	>92	gasoline, diesel	[45]
2016	Cu mesh	dodecanthiol (DDT)	thermal oxidation	95	dodecane, hexadecane	[46]
